# Beta-Caryophyllene Exhibits Anti-Proliferative Effects through Apoptosis Induction and Cell Cycle Modulation in Multiple Myeloma Cells

**DOI:** 10.3390/cancers13225741

**Published:** 2021-11-16

**Authors:** Federica Mannino, Giovanni Pallio, Roberta Corsaro, Letteria Minutoli, Domenica Altavilla, Giovanna Vermiglio, Alessandro Allegra, Ali H. Eid, Alessandra Bitto, Francesco Squadrito, Natasha Irrera

**Affiliations:** 1 Department of Clinical and Experimental Medicine, University of Messina, Via C. Valeria Gazzi, 98125 Messina, Italy; fmannino@unime.it (F.M.); gpallio@unime.it (G.P.); robi.corsaro@libero.it (R.C.); lminutoli@unime.it (L.M.); nirrera@unime.it (N.I.); 2 Department of Biomedical, Dental, Morphological and Functional Imaging Sciences, University of Messina, Via C. Valeria Gazzi, 98125 Messina, Italy; daltavilla@unime.it (D.A.); giovanna.vermiglio1@unime.it (G.V.); 3 Department of Human Pathology in Adulthood and Childhood, University of Messina, Via C. Valeria Gazzi, 98125 Messina, Italy; alessandro.allegra@unime.it; 4 Department of Basic Medical Sciences, College of Medicine, QU Health, Qatar University, 2713 Doha, Qatar; ali.eid@qu.edu.qa; 5 Biomedical and Pharmaceutical Research Unit, QU Health, Qatar University, 2713 Doha, Qatar

**Keywords:** beta-caryophyllene, cannabinoid receptor 2, multiple myeloma, apoptosis, Wnt/β-catenin

## Abstract

**Simple Summary:**

Multiple myeloma (MM) is a malignant B-cell neoplasm characterized by the uncontrolled proliferation of plasma cells. MM cells highly express cannabinoid type 2 receptors (CB2Rs), and previous studies have already demonstrated that the Cannabis plant and its derivatives may have anti-emetic as well as anti-neoplastic effects. In the present study, β-caryophyllene (BCP), a natural CB2R agonist, was evaluated for its anti-proliferative and anti-cancer effects. BCP was able to induce the apoptotic mechanism by activating the molecules involved in triggering apoptosis, such as Bax and caspase 3, and it reduced the anti-apoptotic protein Bcl-2; BCP also regulated cell proliferation through sophisticated crosstalk between Akt, β-catenin, and cyclin D1/CDK 4-6 in a concentration-dependent manner. These effects were counteracted by AM630, a CB2R antagonist, thus showing that BCP acts through CB2R. The data obtained so far demonstrate that BCP, thanks to its anti-proliferative effects, might represent an interesting additional therapeutic approach to improve anti-myeloma therapy.

**Abstract:**

Cannabinoid receptors, which are widely distributed in the body, have been considered as possible pharmacological targets for the management of several tumors. Cannabinoid type 2 receptors (CB2Rs) belong to the G protein-coupled receptor family and are mainly expressed in hematopoietic and immune cells, such as B-cells, T-cells, and macrophages; thus, CB2R activation might be useful for treating cancers affecting plasma cells, such as multiple myeloma (MM). Previous studies have shown that CB2R stimulation may have anti-proliferative effects; therefore, the purpose of the present study was to explore the antitumor effect of beta-caryophyllene (BCP), a CB2R agonist, in an in vitro model of MM. Dexamethasone-resistant (MM.1R) and sensitive (MM.1S) human multiple myeloma cell lines were used in this study. Cells were treated with different concentrations of BCP for 24 h, and a group of cells was pre-incubated with AM630, a specific CB2R antagonist. BCP treatment reduced cell proliferation through CB2R stimulation; notably, BCP considerably increased the pro-apoptotic protein Bax and decreased the anti-apoptotic molecule Bcl-2. Furthermore, an increase in caspase 3 protein levels was detected following BCP incubation, thus demonstrating its anti-proliferative effect through apoptosis activation. In addition, BCP regulated AKT, Wnt1, and beta-catenin expression, showing that CB2R stimulation may decrease cancer cell proliferation by modulating Wnt/β-catenin signaling. These effects were counteracted by AM630 co-incubation, thus confirming that BCP’s mechanism of action is mainly related to CB2R modulation. A decrease in β-catenin regulated the impaired cell cycle and especially promoted cyclin D1 and CDK 4/6 reduction. Taken together, these data revealed that BCP might have significant and effective anti-cancer and anti-proliferative effects in MM cells by activating apoptosis, modulating different molecular pathways, and downregulating the cell cycle.

## 1. Introduction

Multiple myeloma (MM) is a malignant B-cell neoplasm characterized by monoclonal plasma cell proliferation in the bone marrow. Among the hematologic tumors, MM is the second-most frequent malignancy worldwide, with over 30,000 cases of MM reported in the United States in 2019 [1]. One of the main hallmarks of MM is the uncontrolled proliferation of clonal plasma cells, which is responsible for its malignancy and possible invasion [2]. This uncontrolled proliferation is mainly due to the dysregulation of the cell cycle, which contributes to the progression of the disease and worsens the prognosis. Some patients may become refractory to the current therapies, which are mainly based on the use of proteasome inhibitors, bisphosphonates, corticosteroids, immunosuppressant drugs, and peripheral blood stem cell transplantation; for this reason, MM is still an incurable cancer [3], and various efforts are devoted to discovering new therapeutic approaches. In this context, previous studies have shown that cannabinoid type 2 receptors (CB2Rs) are highly expressed in B-cells, which are plasma cell (PCs) precursors, and in hematopoietic cells [4,5]. In addition, MM cell lines and primary MM cells highly express CB2Rs, suggesting significant expression of CB2Rs in B PCs as well [6]. Interestingly, some studies have indicated that immune cells are able to secrete 2-arachidonoyl glycerol (2-AG), an endocannabinoid that acts as an agonist of cannabinoid receptors [7,8,9]. Several studies have demonstrated the anti-cancer activity of cannabinoids [10,11,12,13], which is mainly ascribed to cell proliferation arrest, selective apoptosis induction, cell cycle modulation, and tumor growth inhibition [14,15]. In particular, a previous study demonstrated that cannabinoid derivatives are able to reduce the cell viability of the MM cell line and primary MPCs collected from high-risk MM patients; interestingly, this anti-proliferative effect was selective toward cancer cells and not normal healthy cells [10].

The high selectivity of cannabinoid derivatives assumes an important translational significance since the available chemotherapeutic agents are not specific to cancer cells and are responsible for a great number of adverse events.

Beta-caryophyllene (β-caryophyllene, BCP) is a non-psychoactive sesquiterpene extracted from Copaifera spp and Cannabis spp with significant antioxidant, anti-inflammatory, chemo-preventive, neuroprotective, and anti-proliferative effects [16,17,18]. BCP has been approved by the Food and Drug Administration (FDA) as a food additive, taste enhancer, and flavoring agent, and it could be used as a nutraceutical and a dietary supplement [16,19]. However, BCP is poorly aqueous-soluble and is sensitive to light, oxygen, humidity, and high temperatures; for this reason, its bioavailability may be affected, thus reducing its pharmacologic activity [20]. In fact, several delivery systems have been developed to overcome this significant limitation and improve both BCP stability and bioavailability [21,22,23] so that this promising compound could be used in future clinical practice.

BCP selectively binds CB2R [24,25], and as a result, it does not induce any psychoactive effects related to CB1 receptor binding. This mechanism of action is responsible for the pharmacological effects of BCP, and its anti-cancer activity is mainly based on cell survival protein inhibition, cell cycle modulation, and apoptosis activation [13].

In the light of the cannabinoid’s anti-cancer effects, evidenced by high CB2R expression in myeloma cells, the aim of this study was to evaluate the effects of BCP, a CB2R agonist, in dexamethasone-resistant (MM.1R) and sensitive (MM.1S) human MM cell lines.

## 2. Materials and Methods

### 2.1. Cell Cultures

MM.1S (steroid-based therapy-resistant) and MM.1R (dexamethasone-resistant), human B lymphoblasts obtained from the peripheral blood of a patient affected by MM, were provided by ATCC^®^ CRL-2974^™^ and ATCC^®^ CRL-2975^™^ (ATCC Manassas, Manassas, VA, USA), respectively. Both cell cultures were plated in RPMI-1640 media (ATCC Manassas, Manassas, VA, USA) with 2 mM L-glutamine, 1 mM sodium pyruvate, 1% antibiotic mixture (Sigma-Aldrich, St. Louis, MO, USA), and 10% fetal bovine serum (FBS) (ATCC Manassas, Manassas, VA, USA) in a humidified incubator at 37 °C with a percentage of 5% CO_2_. In addition, RPMI 1788 cells (ATCC^®^ CCL-156™; ATCC Manassas, Manassas, VA, USA), which are human B lymphoblasts obtained from the peripheral blood of a healthy donor, were cultured in RPMI-1640 medium supplemented with 20% FBS (ATCC Manassas, Manassas, VA, USA) and a 1% antibiotic mixture (Sigma-Aldrich, St. Louis, MO, USA) in a humidified incubator at 37 °C with a percentage of 5% CO_2_.

### 2.2. Cell Treatment

MM.1S and MM.1R were placed in culture using 6-well plates with a density of 2.5 × 10^5^ cells/well; cells were treated with BCP (Sigma-Aldrich, St. Louis, MO, USA; purity >80%) at concentrations of 50 and 100 μM for 24 h. At the end of BCP treatment, cells were collected to perform fluorescein diacetate/propidium iodide (FDA/PI) staining, molecular evaluations, and immunofluorescence. In addition, a group of MM.1S and MM.1R cells were treated with AM630 (Tocris Bioscience, Oxford, UK), a CB2 receptor antagonist, at a concentration of 100 nM 2 h before BCP treatment.

### 2.3. FDA/PI Staining

FDA/PI staining (Sigma-Aldrich, St. Louis, MO, USA) was used to evaluate MM.1S and MM.1R cell viability. FDA stock solution was prepared by dissolving 5 mg of FDA in 1 mL of acetone, and a PI stock solution was prepared by dissolving 2 mg of PI in 1 mL of phosphate-buffered saline (PBS). The FDA/PI staining solution was prepared by adding 8 μL of FDA (5mg/mL) and 50 μL of PI (2 mg/mL) in 5 mL of culture medium without FBS. Cells were seeded at a density of 5 × 10^5^ cells/well in a 24-well plate and treated with 50 and 100 μM BCP. The culture medium was removed after 24 h, and cells were stained with the FDA/PI staining solution for 5 min at room temperature in the dark. Viable cells were observed with a fluorescence microscope. The quantification of positive cells was performed with ImageJ software for Windows (Softonic, Barcelona, Spain).

### 2.4. MTT Assay

An MTT assay was carried out to evaluate cancer cell viability following BCP treatment. MM.1S, MM.1R, and RPMI 1788 were seeded in a 96-well plate at a density of 2 × 10^5^ cells/well for 24 h. The day after, cells were treated with doubling concentrations of BCP (6.25, 12.5, 25, 50, 100, and 200 μM) for 24 h in order to evaluate the cytotoxic effect, as previously described [25].

### 2.5. Trypan Blue Assay

Trypan blue dye was used to quantify the comparative number of live and dead cells. Cells were collected and centrifuged at 1200 rpm for 5 min. The obtained cell pellets were resuspended in 1 mL of fresh medium. The suspension and a 0.4% trypan blue/PBS solution were mixed in a 1:1 ratio. Ten microliters of this mixture was loaded on a hemocytometer and visualized with an optical microscope. The percent viability was determined using the following formula:

% viable cells = [1.00 − (Number of blue cells ÷ Number of total cells)] × 100

### 2.6. Measurements of Proteins by Enzyme-Linked Immunosorbent Assay (ELISA)

CDK4, CDK6, and Wnt1 levels were evaluated in the cell lysates, using the respective enzyme-linked immunosorbent assay (ELISA) kits (LSBio, Seattle, WA, USA, or MyBioSource, San Diego, CA, USA), following the instructions reported by the manufacturer [26].

### 2.7. Western Blot Analysis

After 24 h of BCP treatment, cells were collected, and the protein expressions of phospho-β-catenin, phospho-Akt, Bax, caspase-3 (Cell Signaling, Danvers, MA, USA), cyclin D1 (Gentex, Irvine, CA, USA), and Bcl-2 (Abcam, Cambridge, UK) were evaluated, as previously described in detail [27,28].

### 2.8. Immunofluorescence Staining

MM.1S and MM.1R were seeded onto glass coverslips, processed for immunofluorescence following 24 h of BCP treatment, and photographed according to the techniques previously described in detail [29].

### 2.9. Statistical Analysis

All results are expressed as means ± standard error of the mean (SEM). The reported results are the means of three experiments. In order to guarantee reproducibility, all assays were performed in duplicate. Statistical analysis was conducted using one-way ANOVA with Tukey’s post hoc test for intergroup comparisons. A *p*-value less than 0.05 was considered significant. Graphs were prepared using GraphPad Prism software (Version 8.0 for macOS, San Diego, CA, USA).

## 3. Results

### 3.1. BCP Reduces Cancer Cells Viability

**Figure 1:**
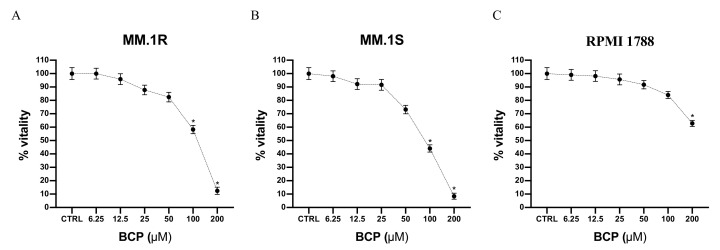
Cell viability evaluated in MM.1R (**A**), MM.1S (**B**), and RPMI 1788 (**C**) cell lines treated with BCP using MTT assays. Values are expressed as percentages of viability reduction compared with control cells. The data are expressed as means ± SEM; *n* = 3 experiments; * *p* < 0.05 vs. Ctrl.

Cell viability was evaluated by incubating MM.1S, MM.1R, and RPMI 1788 cell lines with increasing concentrations of BCP, ranging from 6.25 μM to 200 μM. The results of the MTT assay showed that MM.1R cell viability was reduced when cells were treated with BCP at concentrations of 25 μM to 200 μM; in particular, cell viability was reduced to about 80% when BCP was used at a concentration of 50 μM and to 50% with 100 μM BCP following 24 h of treatment (Figure 1A). As shown in Figure 1B, reductions in cell viability of about 70% and 50% were observed when MM.1S cells were treated with 50 and 100 μM BCP, respectively. Moreover, BCP treatment did not affect the cell viability of RPMI1788 cells, demonstrating its selective antiproliferative effect on MM cells (Figure 1C).

**Figure 2:**
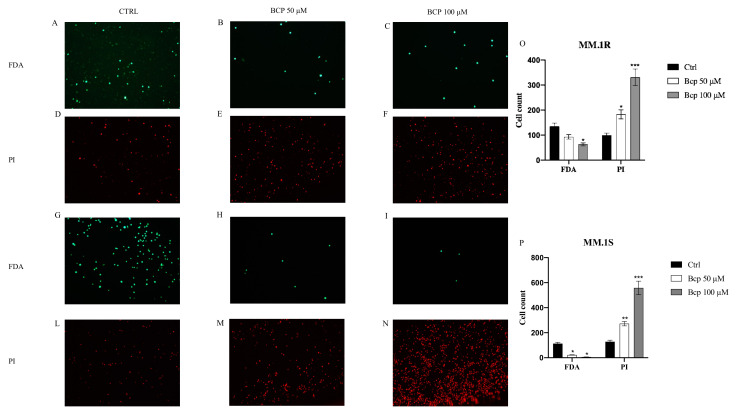
The figure represents the apoptotic process evaluated by FDA/PI staining in MM.1R and MM.1S cell lines treated with BCP. In panels (**A**–**C**) and (**G**–**I**), green color reaction indicates viable MM.1R and MM.1S cells, respectively; in panels (**D**–**F**) and (**L**–**N**), red reaction indicates MM.1R and MM.1S cells that underwent apoptosis, respectively. Panels (**O**) and (**P**) show the cells counts in MM.1R and MM.1S. The data are expressed as means ± SEM; *n* = 3 experiments; * *p* < 0.05 vs. Ctrl. ** *p* < 0.001 vs. Ctrl. *** *p* < 0.0001 vs. Ctrl.

Images obtained from the FDA/PI staining also showed cell viability: live cells were bright green and nonviable cells were red. Notably, untreated MM.1S cells stained with FDA showed bright fluorescence, but a low level of fluorescence was observed with PI labeling; MM.1R cells treated with BCP at concentrations of 50 and 100 μM for 24 h showed few cells stained with FDA but many nuclei stained with PI (Figure 2A–F). Overlapping results were obtained in the MM.1S cell line (Figure 2G–N). The graphs presented in Figure 2 O, P represent the cell counts of MM.1R and MM.1S positive cells. BCP at a concentration of 50 μM increased the number of PI-positive cells in both cell lines (*p* < 0.05 vs. CTRL) and reduced the number of positive FDA cells only in MM.1S cells (*p* < 0.05 vs. CTRL). BCP at a concentration of 100 μM significantly increased the number of PI-positive cells (*p* < 0.0001 vs. CTRL) and strongly reduced the number of FDA-positive cells in both cell lines (*p* < 0.05 vs. CTRL).

**Figure 3:**
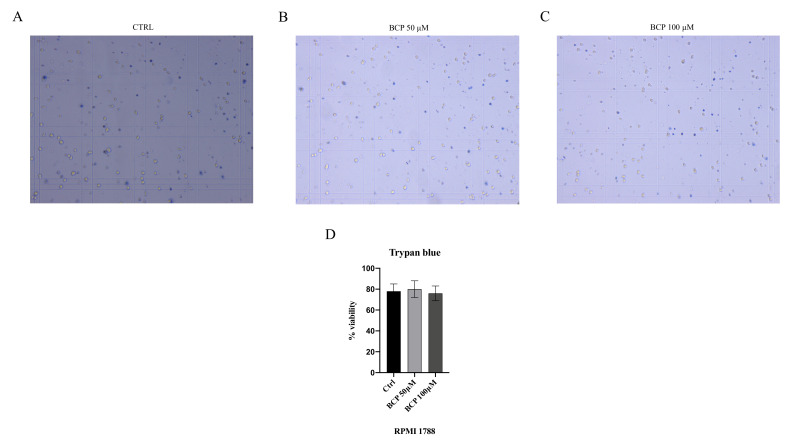
The figure represents the trypan blue staining in RPMI 1788 cells treated with BCP. In panels (**A**–**C**), blue color reaction indicates RPMI 1788 cells that underwent apoptosis. Panel (**D**) shows the percentage of viable cells. The data are expressed as means ± SEM; *n* = 3 experiments.

In addition, a trypan blue assay was performed to confirm the selective antiproliferative effect of BCP on the MM.1S and MM.1R cell lines. The RPMI 1788 cell line, used as normal cells, was treated with BCP at concentrations of 50 and 100 μM for 24 h, thus demonstrating that BCP did not affect the proliferation of normal cells (Figure 3) and confirming the MTT results and BCP-selective effect in the MM cell lines.

### 3.2. BCP Treatment Induces Apoptotic Pathways in MM.1S and MM.1R Cell Lines

**Figure 4:**
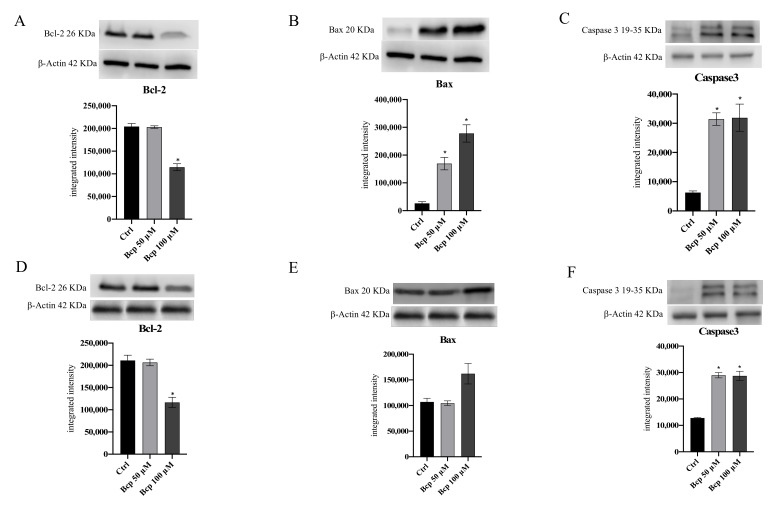
The graphs represent Bcl-2 (**A**), Bax (**B**), and caspase 3 (**C**) protein expression in MM.1S cells and protein expression of Bcl-2 (**D**), Bax (**E**), and caspase3 (**F**) in MM.1R cells treated with BCP. The data are expressed as means ± SEM; *n* = 3 experiments; * *p* < 0.05 vs. Ctrl.

Bcl-2, Bax, and caspase-3 protein expression were studied using Western blot analysis to evaluate whether BCP induces the apoptotic pathway in MM.1S (Figure 4A–C, Figure S1) and MM.1R (Figure 4D–F, Figure S1) cancer cells. BCP significantly increased caspase-3 and Bax, whereas it reduced Bcl-2 expression, compared with untreated cells in both MM.1S and MM.1R following 24 h of treatment especially at a concentration of 100 μM, thus indicating that BCP induced the apoptotic process in MM cancer cells (*p* < 0.05 vs. CTRL; Figure 4).

### 3.3. BCP Has A Significant Anti-Proliferative Effect through Akt and Wnt/β-Catenin Modulation

**Figure 5:**
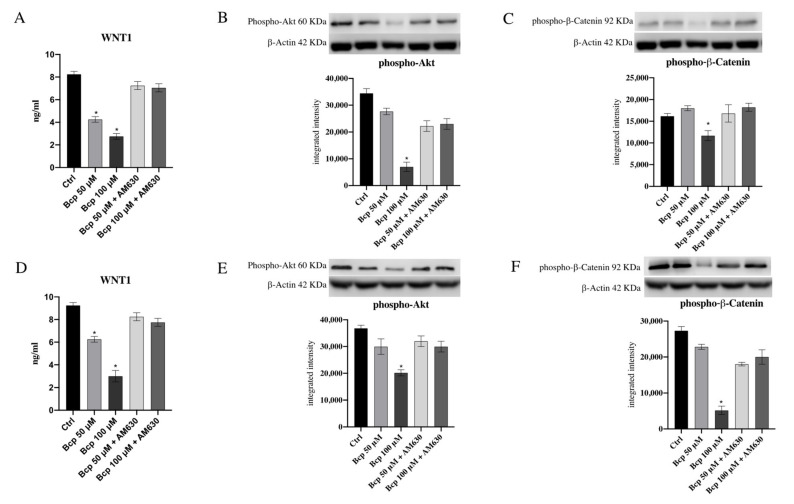
The graphs represent protein levels of Wnt1 (**A**), p-Akt (**B**), and p-β-Catenin (**C**) in MM.1S cells and Wnt1 (**D**), p-Akt (**E**), and p-β-Catenin (**F**) protein levels in MM.1R cells treated with BCP. The data are expressed as means ± SEM; *n* = 3 experiments; * *p* < 0.05 vs. Ctrl.

Wnt1 protein levels as well as p-Akt and β−catenin protein expression were evaluated to investigate BCP’s anti-proliferative effects in MM.1S and MM.1R cancer cells. BCP treatment caused a marked reduction in Wnt1 in the MM.1S cell line (*p* < 0.05 vs. CTRL; Figure 5), particularly at a concentration of 100 μM. Similar results were obtained in the MM.1R cancer cell line: BCP significantly reduced Wnt1 following 24 h of treatment compared with untreated cells (*p* < 0.05 vs. CTRL; Figure 5). In addition, both cell lines treated with BCP at a concentration of 100 μM showed a significant decrease in p-Akt and β-catenin protein expression, confirming that BCP treatment may have an anti-proliferative effect through Wnt1, p-Akt, and β-catenin reduction (*p* < 0.05 vs. CTRL; Figure 5 and Figures S2–S5). Wnt1, p-Akt, and β-catenin reduction was reversed by the treatment with the CB2R antagonist AM630, which abrogated BCP’s effects, thus demonstrating that BCP’s mechanism of action was related to CB2 receptor modulation.

**Figure 6:**
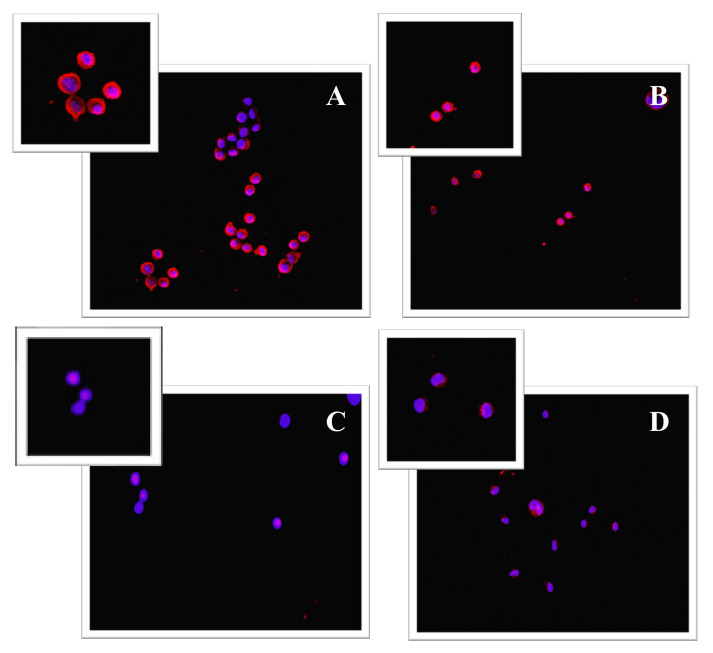
Panel of immunofluorescence staining for β-catenin (red fluorescence). β-catenin fluorescence pattern in control cells, MM.1S (**A**), and MM.1R (**B**) is mainly distributed at the perinuclear and plasma membrane level, as clearly shown in the magnifications in the left corners; MM.1 S cells treated with BCP for 24 h showed a strong reduction in β-catenin staining (**C**); BCP treatment for 24 h caused a significant β-catenin fluorescence reduction in MM.1R treated cells (**D**).

The reduction in β-catenin activation following BCP treatment was also observed in immunofluorescence. Control cells showed positive staining for β-catenin; this staining pattern was appreciable around the nuclei and at the plasma membrane level (Figure 6A,B). The images in Figure 6C (Figure S6) and D show a very low β-catenin staining pattern, thus confirming BCP’s efficacy in reducing β-catenin activation.

### 3.4. BCP Anti-Proliferative Effect Is Carried out through Cell Cycle Inhibition

**Figure 7:**
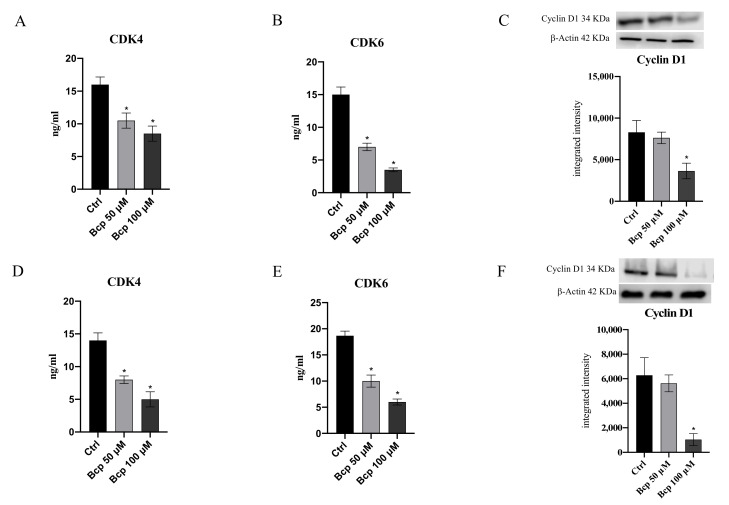
The graphs represent CDK4 (**A**), CDK6 (**B**), and cyclin D1 (**C**) protein levels in MM.1S cells. CDK4 (**D**), CDK6 (**E**), and cyclin D1 (**F**) protein levels in MM.1R cells treated with BCP. The data are expressed as means ± SEM; *n* = 3 experiments; * *p* < 0.05 vs. Ctrl.

The hypothesis that BCP might exert an anti-proliferative effect in MM cancer cells was further confirmed by the results obtained for cyclin D1 expression and the levels of its kinases, CDK 4 and 6. BCP treatment, particularly at a concentration of 100 μM, significantly reduced CDK4 and CDK6 levels both in MM.1S and MM.1R cells compared with untreated cells (*p* < 0.05 vs. CTRL; Figure 7). As expected, cyclin D1 protein expression was downregulated when MM.1S and MM.1R cells were treated with BCP at a concentration of 100 μM following 24 h of treatment (*p* < 0.05 vs. CTRL; Figure 7 and Figures S6 and S7).

## 4. Discussion

In the past years, several efforts have been made by the scientific community to characterize the role of the ECS in several body areas and to understand if cannabinoid receptors might be considered as effective therapeutic targets. The use of the cannabis plant and its derivatives might represent a new therapeutic window for the management of diseases for which there is no effective therapy, such as cancers, and have a significant impact on medicine and the global economy. Cannabinoids have shown anti-emetic effects in cancer patients as well as significant anti-neoplastic effects in solid tumors, such as glioma and breast cancer [30,31,32]. The exact mechanism of action is not completely understood, but the therapeutic approach is mainly based on the wide distribution of cannabinoid receptors. MM cells express cannabinoid receptors, as demonstrated by flow cytometry analysis [10,33], and experimental studies have demonstrated that both cannabidiol and different cannabinoid derivatives induce apoptosis in MM cell lines through a caspase-dependent mechanism [7,10].

In the present experimental setting, BCP, a natural CB2R agonist, was evaluated for its anti-proliferative and anti-neoplastic effects in MM.1S and MM.1R cells; in particular, MM.1R cells respond less to chemotherapy and represent a condition observed in cancer patients in advanced stages of the disease [34]. Our team already revealed that BCP reduced cell viability in glioma cell lines and glioma-derived stem-like cells, thus demonstrating that BCP might be used in conditions of resistance [13]. BCP is a safe compound; in fact, it did not affect healthy cells, such as human gingival fibroblasts and human oral mucosa epithelial cells, as already demonstrated in our previous study [25]; in addition, another CB2 agonist, WIN-55,212–2 mesylate, was not only effective in reducing cancer cells proliferation but was also selective toward cancer cells and not control cells (healthy cells) [10], thus supporting the hypothesis that BCP might be used in the clinical practice, affecting cancer cells and remaining safe for normal cells. In accordance with our experimental hypothesis, it has been previously confirmed that cannabinoid use may selectively stimulate apoptosis in MM cells through a caspase-2-dependent mechanism, but what is even more interesting is that cell death was only activated in MM cells and did not affect normal cells; moreover, cannabinoid-induced apoptosis was inhibited by blocking CB2R [10,33].

BCP also proved effective in MM.1S cells and the more resistant MM.1R cell line at concentrations of 50 μM and 100 μM, thus reducing the cell viability of cancer cells and not affecting normal cells, as demonstrated by the MTT assay and trypan blue staining; in particular, the fluorescent marking with FDA confirmed the MTT results and demonstrated that BCP significantly reduced the number of viable cells. This antiproliferative effect is mainly due to the activation of apoptosis as a cell death mechanism. In fact, the triggering of Bax and caspase-3 following BCP treatment in both cell lines pointed out that BCP anti-neoplastic activity might be ascribed to a caspase-dependent mechanism of cell death and p53-mediated apoptosis through Bax stimulation. In addition, Bcl-2, which usually promotes cell survival and is considered an anti-apoptotic protein [35], was significantly reduced in treated cancer cells in favor of apoptotic markers, thus confirming BCP’s pro-apoptotic effect. Cannabinoids were able to induce apoptosis in melanoma, glioma, breast cancer, and MM cells through a molecular mechanism that provides for Akt modulation, which is one of the most strongly involved pathways in response to cannabinoid receptor stimulation [15,36].

Phosphatidylinositol-3-kinase (PI3K)/Akt and the mammalian target of rapamycin (mTOR) signaling pathway, which controls cell proliferation, is abnormally activated in several cancers and also in MM patients [37]; therefore, a drug that inhibits this pathway might be effective in the treatment of MM. BCP treatment markedly reduced Akt expression, particularly at a concentration of 100 μM, in both cell lines compared with untreated cells, thus providing an important translational relevance since Akt overexpression often correlates with poor outcomes [38]. PI3K/Akt/mTOR is a sophisticated pathway that is interconnected with other signaling pathways, such as Wnt/β-catenin signaling. Wnt/β-catenin pathway activation is involved in both normal development and aberrant cell proliferation; in fact, a β-catenin increase may exert oncogenic effects in different tumors [39,40]. BCP’s anti-proliferative effect was established by the results obtained from Wnt1 and β-catenin expression: a significant decrease was observed in the treated MM.1R and MM.1S cell lines compared with untreated cancer cells. These anti-proliferative effects were abrogated when MM.1 cells were treated with both BCP and the CB2R antagonist AM630, thus demonstrating that BCP’s mechanism of action is strictly related to CB2R modulation since AM630 antagonizes this specific receptor. MM.1 cells treated with BCP showed a significant reduction of the positive fluorescence of β-catenin compared with controls (tumor cells). These data indicate that BCP’s anti-proliferative effect may be due to the complex modulation of the Akt and Wnt/β-catenin pathways through the essential stimulation of the apoptotic mechanism.

**Figure 8:**
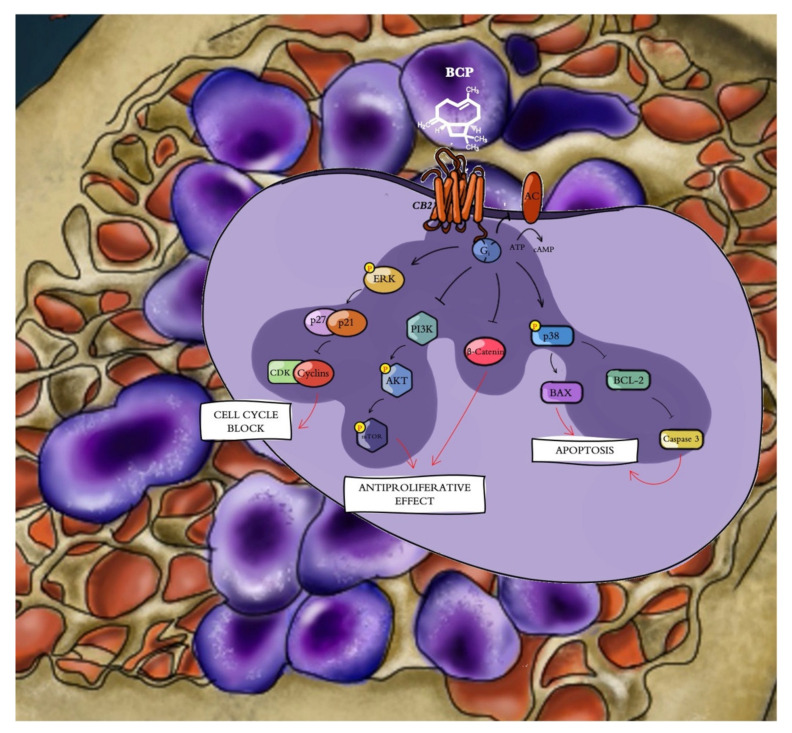
Beta-caryophyllene (β-caryophyllene, BCP) selectively binds cannabinoid type 2 receptor (CB2R), thus inducing an antiproliferative effect through (i) cell cycle modulation by reducing cyclin D1 and Cdk 4/6 expression, (ii) apoptosis activation by increasing Bax and caspase 3 and reducing Bcl-2 expression, and (iii) Akt and β-catenin inhibition.

The cyclin D1 gene is a target for β-catenin and is accountable for the progression of cells into the proliferative stage of the cell cycle [41]. In fact, β-catenin-mediated signaling depends on its accumulation and consequent translocation into the nucleus, where it regulates gene transcription. Increased β-catenin levels were associated with malignancies, and this increase is considered one of the features of MM, thus promoting tumor progression through cell cycle activation [42]. The cell cycle is a process that is regulated by different cyclins and their CDKs; the likelihood of developing cancer dramatically increases when the precise balance between cyclins and CDKs is impaired [43]. One of the main alterations observed in cancer concerns cyclin D and CDK4/6 overexpression, and in particular, cyclin D alteration is one of the key hallmarks of MM [44,45]. For this reason, targeting these cell cycle regulators may represent a promising therapeutic approach for the management of myeloma. Surprisingly, in our experimental model, we observed that BCP treatment induced the reduction of cyclin D1 and its kinases, CDK4 and CDK6, in both the MM.1S and MM.1R cell lines compared with untreated cells, further demonstrating BPC’s anti-proliferative effects through cell cycle modulation, probably as a consequence of β-catenin reduction (Figure 8).

## 5. Conclusions

MM is considered an incurable plasma cell cancer; blocking cell proliferation and consequently cell progression in terms of invasion may represent an interesting approach to improve anti-myeloma therapy. On one hand, BCP was able to induce the apoptotic mechanism, activating the molecules involved in apoptosis; moreover, BCP regulated cell proliferation through sophisticated crosstalk between Akt, β-catenin, and cyclin D/CDK 4/6 in a concentration-dependent manner. However, in vivo experimental approaches should be developed to confirm the results described so far and demonstrate that BCP might represent an interesting alternative or additional therapeutic approach to conventional chemotherapy for the treatment of multiple myeloma.

## Data Availability

The datasets generated for this study are available on request to the corresponding author.

## Supplementary Materials

The following are available online at https://www.mdpi.com/article/10.3390/cancers13225741/s1, Figure S1: Original western blots for Figure 4A–F, Figure S2: Original Western blots for Figure 5B, Figure S3: Original Western blots for Figure 5C, Figure S4: Original Western blots for Figure 5E, Figure S5: Original Western blots for Figure 5F, Figure S6: Original Western blots for Figure 7C, Figure S7: Original figure blots for Figure 7F.
