# Correction: Mannino et al. Beta-Caryophyllene Exhibits Anti-Proliferative Effects through Apoptosis Induction and Cell Cycle Modulation in Multiple Myeloma Cells. *Cancers* 2021, *13*, 5741

**DOI:** 10.3390/cancers15215250

**Published:** 2023-11-01

**Authors:** Federica Mannino, Giovanni Pallio, Roberta Corsaro, Letteria Minutoli, Domenica Altavilla, Giovanna Vermiglio, Alessandro Allegra, Ali H. Eid, Alessandra Bitto, Francesco Squadrito, Natasha Irrera

**Affiliations:** 1 Department of Clinical and Experimental Medicine, University of Messina, Via C. Valeria Gazzi, 98125 Messina, Italygpallio@unime.it (G.P.);; 2 Department of Biomedical, Dental, Morphological and Functional Imaging Sciences, University of Messina, Via C. Valeria Gazzi, 98125 Messina, Italy; 3 Department of Human Pathology in Adulthood and Childhood, University of Messina, Via C. Valeria Gazzi, 98125 Messina, Italy; 4 Department of Basic Medical Sciences, College of Medicine, QU Health, Qatar University, Doha 2713, Qatar; 5 Biomedical and Pharmaceutical Research Unit, QU Health, Qatar University, Doha 2713, Qatar

## Correction of Western Blot Images for Figures 4 and 7

In the original publication [1], errors were identified in the published Figures 4 and 7. The original raw data of western blots could not be retrieved and therefore the experiments were redone to generate the corrected Figures 4 and 7. The supplementary files for western blots were also updated to include the raw data.

For Figure 4A–F, the blots of samples ctrl, BCP 50, and BCP 100 for the targets Bcl2, Bax, Caspase 3, Cyclin D1, and the β actins are re-analyzed. All of the blot images and integrated intensity are updated for the figure. 

For Figure 7, the blots of samples ctrl, BCP 50, and BCP 100 for the targets Cyclin D1 and the β actins are re-analyzed. The subfigures C and F are updated with the new information.

It was confirmed that the study’s original conclusions are still valid and unchanged. 

The corrected Figure 4 and Figure 7 appear below:

**Figure 4:**
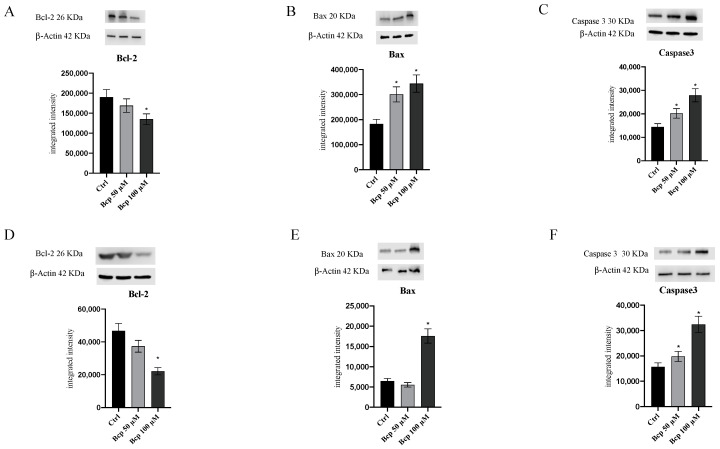
The graphs represent Bcl-2 (**A**), Bax (**B**), and Caspase 3 (**C**) protein expression in MM.1S cells and protein expression of Bcl-2 (**D**), Bax (**E**), and Caspase3 (**F**) in MM.1R cells treated with BCP. The data are expressed as means ± SEM; *n* = 3 experiments; * *p* < 0.05 vs. Ctrl. The original western blots can be found in the Supplementary Materials.

**Figure 7:**
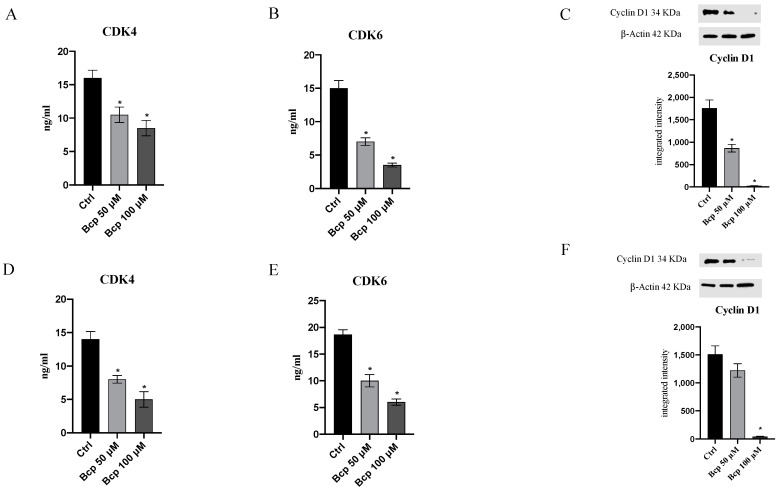
The graphs represent CDK4 (**A**), CDK6 (**B**), and cyclin D1 (**C**) protein levels in MM.1S cells. CDK4 (**D**), CDK6 (**E**), and cyclin D1 (**F**) protein levels in MM.1R cells treated with BCP. The data are expressed as means ± SEM; *n* = 3 experiments; * *p* < 0.05 vs. Ctrl. The original western blots can be found in the Supplementary Materials.

The authors apologize for any inconvenience caused and state that the scientific conclusions are unaffected. This correction was approved by the Academic Editor. The original publication has also been updated.
