# Supplementary material for: Beta-Caryophyllene Exhibits Anti-Proliferative Effects through Apoptosis Induction and Cell Cycle Modulation in Multiple Myeloma Cells
Source: Cancers (Basel). 2021 Nov 16;13(22):5741. doi: 10.3390/cancers13225741 (PMC8616110; doi:10.3390/cancers13225741)
Supplement: Supplementary file 1 [file cancers-13-05741-s001.zip › cancers-1438998-supplementary.pdf]

Supplementary Materials: Beta-Caryophyllene Exhibits Anti-Proliferative Effects through Apoptosis Induction and Cell Cycle Modulation in Multiple Myeloma Cells

Federica Mannino, Giovanni Pallio, Roberta Corsaro, Letteria Minutoli, Domenica Altavilla, Giovanna Vermiglio, Alessandro Allegra, Ali H. Eid, Alessandra Bitto, Francesco Squadrito and Natasha Irrera

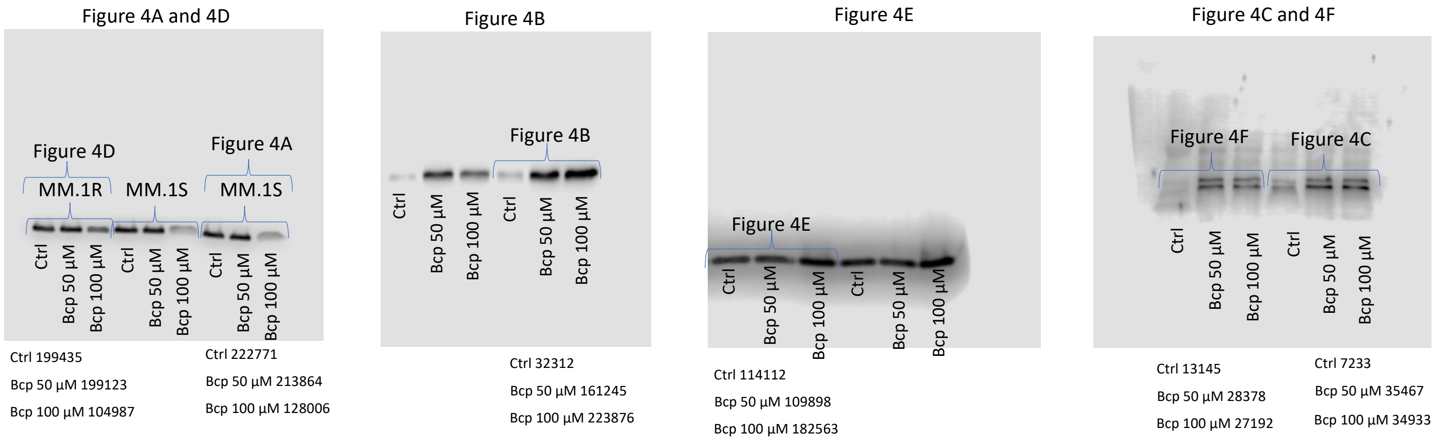

Figure S1. Original western blots for Figure 4**A–F**.

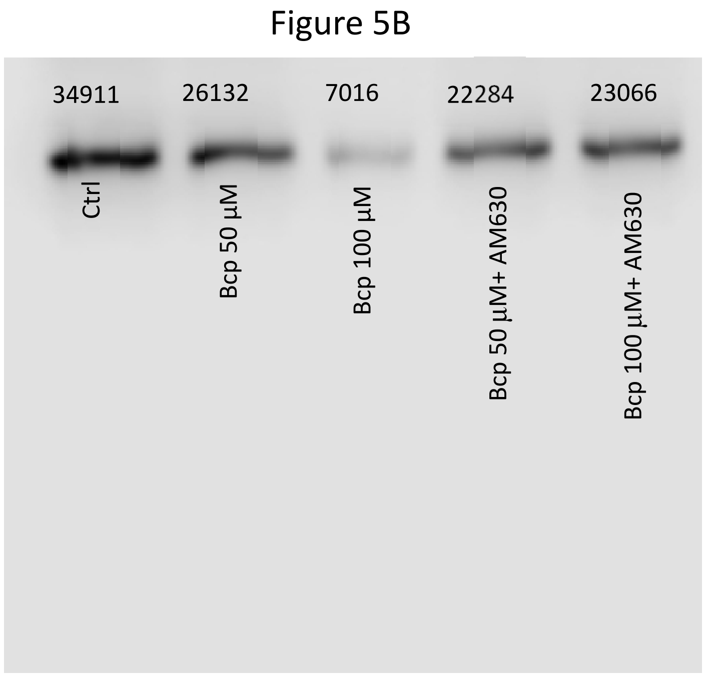

Figure S2. Original Western blots for Figure 5B.

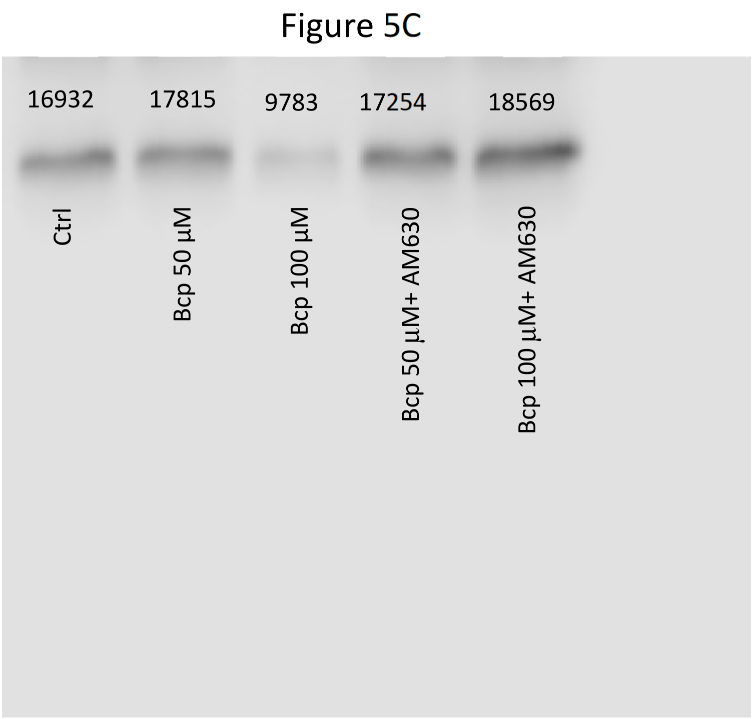

Figure S3. Original Western blots for Figure 5C.

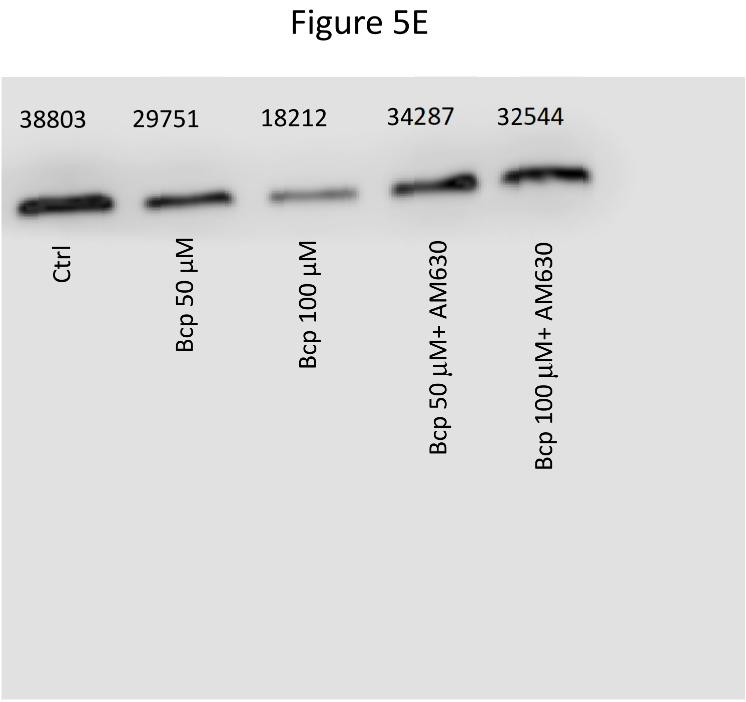

Figure S4. Original Western blots for Figure 5E.

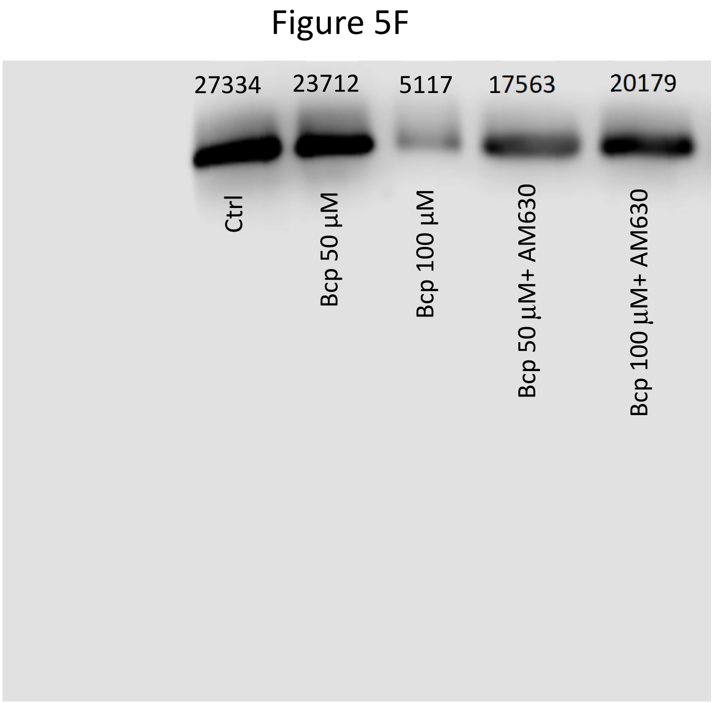

Figure S5. Original Western blots for Figure 5F.

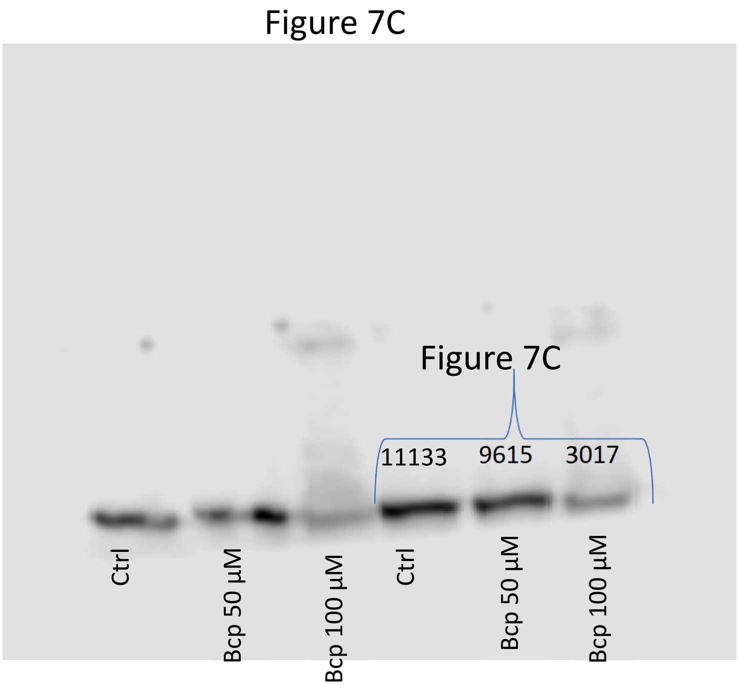

Figure S6. Original Western blots for Figure 7C.

Figure 7F

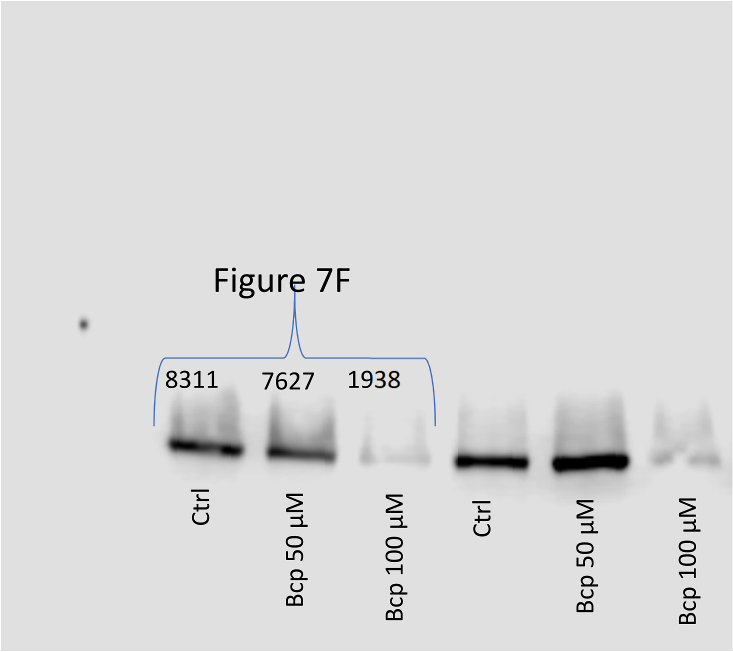

Figure S7. Original figure blots for Figure 7F.
